# Grisel's syndrome associated with mumps: A case report

**DOI:** 10.3389/fped.2022.916538

**Published:** 2022-09-29

**Authors:** Yanrong Shen, Lixia Yang, Xiaoliang Liu, Yawen Xie, Xiaohui Dai, Chuan Wang

**Affiliations:** ^1^Department of Pediatric Cardiology, West China Second University Hospital, Sichuan University, Chengdu, China; ^2^West China Medical School of Sichuan University, Chengdu, China; ^3^The Cardiac Development and Early Intervention Unit, West China Institute of Women and Children's Health, West China Second University Hospital, Sichuan University, Chengdu, China; ^4^Key Laboratory of Birth Defects and Related Diseases of Women and Children (Sichuan University), Ministry of Education Chengdu, Chengdu, China; ^5^Key Laboratory of Development and Diseases of Women and Children of Sichuan Province, West China Second University Hospital, Sichuan University, Chengdu, China; ^6^Department of Ultrasonography, West China Second University Hospital, Sichuan University, Chengdu, China

**Keywords:** Grisel's syndrome, mumps, atlanto-axial subluxation, case report, etiology

## Abstract

Grisel's syndrome (GS) is defined as atlantoaxial rotatory subluxation/fixation not associated with trauma or bone disease, usually following head and neck infection/inflammation or ear, nose, and throat (ENT) surgery. Many conditions could lead to Grisel's syndrome, of which mumps is rarely to be seen. This report discusses a case of GS in children with Type I atlantoaxial joint subluxation and previously diagnosed mumps. A 6-year-old boy who had cervical pain and torticollis for 2 weeks was admitted to our hospital. There was no trauma and he had not had ENT surgery but was diagnosed with mumps 2 weeks previously due to swelling of the left cheek and cervical lymph node. Physical examination and computed tomography confirmed a diagnosis of Grisel's syndrome with an ADI (atlanto-dens interval) of 1.6 mm. The patient then received occipito-mandibular traction for 6 days and recovered. No recurrence was observed at 1 year follow-up. Physicians should raise awareness of this rare complication of mumps to avoid life-threatening neurological impairments owing to Grisel's syndrome.

## Introduction

Grisel's syndrome (GS) is defined as atlantoaxial rotatory subluxation/fixation not associated with trauma or bone disease, usually following head and neck infection/inflammation or ear, nose, and throat (ENT) surgery (1–3). It is a rare disorder that mainly occurs in children. Typical symptoms include torticollis, neck pain, and limited motion of cervical vertebrae. Despite these symptoms, most patients with GS display a good prognosis as long as being diagnosed and treated on time. Recent research documented that patients with GS present a later recovery than those with other atlantoaxial rotatory subluxation/fixation (4). Furthermore, a small number of GS patients may develop neurological impairments while GS progresses, such as quadriplegia or paresthesia (5), which may be permanent, irreversible, and even fatal. Therefore, it is of great importance and clinical significance for clinicians to have a better awareness and recognization of this rare disorder and its underlying etiologies to avoid the possibility of serious consequences. In terms of etiologies, infection of the upper respiratory tract or head and neck regions, ear, nose, and throat (ENT) surgical procedures are common causes of GS (6). Some other rare conditions such as Kawasaki disease (7), cervical tuberculosis (8), infectious mononucleosis (9), and mycoplasma pneumoniae infection (10) have been reported sporadically. However, GS associated with mumps has rarely been documented in the literature.

Mumps, or epidemic parotitis, resulting from mumps virus infection, is a common disease among children that usually presents with a satisfactory prognosis. Several complications, such as orchitis, oophoritis, myocarditis, and meningitis, have been widely recognized and reported (11). Mumps often involves both sides of the cheek, which are dangerous places where inflammation may affect deep tissues around cervical regions and may lead to GS. However, GS has rarely been considered a complication of mumps. To our knowledge, only two cases have been previously described (12, 13).

Given the possibility of later recovery and life-threatening neurological impairments caused by GS, we here report on a case of GS that developed in a child due to mumps in a Chinese population, aiming to enhance and improve the awareness and recognization of this rare complication in patients with mumps.

## Case presentation

**Figure 1:**
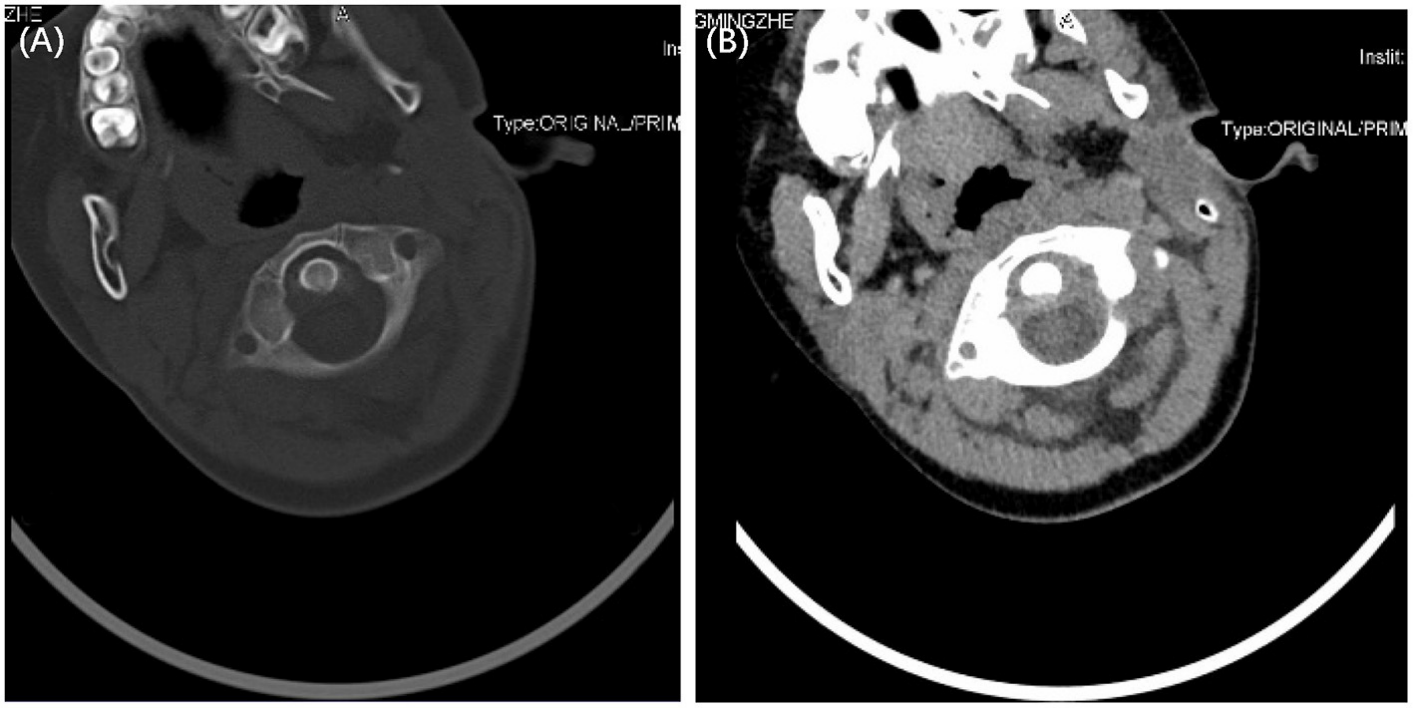
A computed tomography scanning showed Type I atlantoaxial joint subluxation **(A,B)**.

A 6-year-old boy who had cervical pain and torticollis for 2 weeks was admitted to the unit of the pediatric trauma department in our hospital. There was no history of trauma or ENT surgeries. However, 2 weeks previously he had been diagnosed with mumps due to swelling of the left cheek and cervical lymph node. The serum mumps virus-specific IgM antibody test was positive. He then received ibuprofen and intravenous rehydration therapy in a local hospital for 3 days. The swelling was relieved, but the torticollis and neck pain continued. He did not develop a fever either at the beginning or after torticollis emerged. A physical examination revealed stable vital signs on admission. He suffered from torticollis toward the left side and severe pain on attempted reduction, with limited motion of the neck, which had 30 degrees flexion, 20 degrees extension, and 25 degrees active right rotation. No eyelid edema was observed. Tonsils showed no swelling and there were no purulent secretions on the surface. No congestion or redness was found in the pharynx. No rash or hepatosplenomegaly was observed. Other physical signs included severe tenderness in the left neck and shoulder. A neurological examination revealed no positive signs. Bilateral cervical lymph nodes showed no swelling. Blood examination demonstrated the following: white blood cells, 7.52 × 10^9^/L; neutrophils, 61.5%; lymphocytes, 32.2%; hemoglobin, 122 g/L; platelets, 629 × 10^9^/L; C-reactive protein (CRP), 2.3 mg/L. Atypical lymphocytes were not found in the peripheral blood of the patient. A computed tomography (CT) scan confirmed the atlantoaxial rotary subluxation, with an increase in the atlanto-dens interval of 1.6 mm, corresponding to a Type I GS (Figures 1A,B).

According to clinical presentations and CT images, the patient was diagnosed with Grisel's syndrome. Other causes, such as tonsillitis, infectious mononucleosis, and Kawasaki disease were excluded since no related symptoms and signs were found. He was treated with occipito-mandibular traction for 6 days. He retrieved complete movement of the neck and significant pain relief. He was then discharged with a cervical collar and asked to sleep in a pillow-free position for 1 month. During 1 year's follow-up, no recurrence or complications were observed, and X-ray confirmed a complete recovery.

## Discussion

GS is a syndrome of torticollis, neck movement disorders, and neck pain that often occurs in children, usually following infection as well as head and neck surgeries. Its pathogenesis is that atlanto-axial joints lose their stability and normal alignment, leading to neck movement disorders. GS is usually secondary to other disease-related conditions. In 1,830, Charles Bell first reported a case of atlantoaxial joint subluxation following pharyngeal ulceration (2). A hundred years later, Pierre Grisel named this syndrome after reporting two cases of joint subluxation due to pharyngitis (14). Since then, lots of reports on GS have emerged. In 1978, the Fielding–Hawkins classification was proposed to classify atlantoaxial subluxation, which divided it into four different classes according to the degree of atlantoaxial joint rotation and subluxation (5). It is worth mentioning that so far there is no gold standard for the management of atlantoaxial joint rotation and subluxation, although it is agreed that external immobilization alone or followed by surgical fixation is the most used therapy. Anania et al. (15) developed a protocol for the management of AARS according to the major evidence in the literature. They recommended different treatment options in accordance with the duration of GS and Fielding–Hawkins classification, which includes drugs, Philadelphia collar, Halo jacket fixation, and surgical procedures.

To date, the widely recognized etiology of GS mainly refers to infection and surgery. Besides that, a small amount of GS patients develop for no reason. Infection mainly involves the upper respiratory tract or head and neck regions, such as pharyngitis, otitis, mastoiditis, lymphadenitis and retropharyngeal abscess (6), tonsillitis, sinusitis, and submandibular abscess (16). Some rare infections include bronchitis and gastroenteritis (7), acute rheumatic fever (1), Kawasaki disease (7), cervical tuberculosis (8), infectious mononucleosis (9), and mycoplasma pneumoniae infection (10). ENT surgery such as tonsillectomy, adenoidectomy, mastoidectomy, tympanoplasty, uvulectomy, and cochlear implantation (6) also account for a predisposing factor. However, among the many cases of GS, we rarely see reports of GS caused by parotitis.

Here, we report on a 6-year-old GS patient with Type I atlantoaxial joint subluxation following parotitis. This is the first report of GS caused by mumps in China. He presented typical GS symptoms at the very beginning of his parotoid glands starting swelling, and the symptoms progressed after the swelling was alleviated. When he was admitted to our hospital, most of his parotitis-related signs had disappeared, leaving only GS-related symptoms. We gave him continuous traction for 6 days and sent him home after he almost recovered. No recurrence or complications were observed in the follow-up. The GS caused by mumps can be traced back to a case reported by Heikkila in 1937 (12), which contains a few accurate materials that are still of relevance. In 2002, Yasunori (13) presented a 7-year-old girl with Grisel's syndrome complicated with mumps. She developed painful torticollis after 2 days of cervical swelling and a low-grade fever. CT confirmed atlanto-axial subluxation but without corresponding Fielding–Hawkins classification. After receiving a total of 4 weeks' traction and cervical stabilization, she had a complete recovery.

Mumps, also known as epidemic parotitis, is a highly contagious disease caused by a paramyxovirus (11). It is characterized by painful swollen salivary glands, and most of the cases occur in children younger than 15 years (1). Mumps is a self-limiting disease with several complications, such as orchitis, oophoritis, myocarditis, and meningitis (11). GS has rarely been reckoned to be one of them, but there still exists the possibility of GS occurrence among mumps patients. From this point of view, clinicians should be aware of the possibility of developing GS in children with mumps and identify it as early as possible.

There is no clear answer to the pathological mechanism of mumps leading to GS. Grisel (14) believed that the subluxation was caused by the compensatory effect of the body on local inflammation mediated by paravertebral and cervical muscle spasms. Later, Wilson (1) proposed that any condition that resulted in hyperemia and pathologic relaxation of the transverse ligament of the atlantoaxial joint could be the main reason. Parke (3) described a liquid circulation flow to explain why local inflammation would lead to unproper relaxation of the transverse ligament. Pharyngovertebral veins traverse the prevertebral fascia and drain into periodontoidal plexuses, finally emptying into the upper cervical epidural venous sinuses. Through anastomoses between the lymphatic and pharyngovertebral veins, inflammatory exudation may infiltrate around the atlantoaxial ligament, which results in the overdistension of ligaments. Nevertheless, instead of inflammation exudation, some researchers thought spasm of cervical muscles is the key to ligament overdistention (17). It helped Battiata et al. (18) propose a two-hit hypothesis for the development of GS: the first would be the preexisting cervical ligamentous laxity due to immaturity of children's ligaments; the second would be muscle spasm resulting from inflammation. As far as we know, the internal side of the parotid gland is adjacent to the parapharyngeal space, which is a potential space formed by the cervical fascia. Meanwhile, this space is posteriorly bounded by the prevertebral fascia and separated from the retropharyngeal space by only a thin layer. Therefore, we speculate that through these very near layers, exudation caused by mumps can affect the vertebrae and ligaments and thus contribute to joint instability. In addition, the deep parotid lymph nodes also receive lymphatic drainage from the pharyngotympanic tube and the posterior nasopharynx, whereby the inflammatory exudation of the parotid gland may circulate through the lymphatic pathways and lead to the loosening of the transverse ligament through the liquid circulation flow described by Parke (3). This infiltration or the secondary muscle spasm may be the critical pathogenic factor.

Delayed diagnosis of GS has always been troublesome during medical practice. In the past, Fielding had reported an average delay of 11.6 months in GS diagnosis (5). A review published in 2020 outlined that the period has been significantly shortened, but the mean delay of GS was still 33 days (7). Furthermore, recent research has revealed that Grisel's syndrome patients who suffer from a concomitant infection/inflammation in the head and neck region, presented later recoveries than other atlantoaxial rotatory subluxation/fixation. These patients are more likely to present disease persistence or relapse at 3 months follow-up (4). Considering that GS patients may have a late recovery and neurological damage resulting from compression of the vertebral artery or spinal cord, only early detection and timely treatment can benefit patients. Since parotitis is not a common cause of GS and thus may be overlooked, it is important to be fully aware of this condition and its outcome. Meanwhile, for patients infected with mumps, it is necessary to consult a clinician in good time and complete a CT examination if the patient developed neck pain or limited mobility. An early diagnosis leads to early intervention, therefore early recovery can be achieved and serious complications can be avoided. However, patients should be reminded to avoid becoming overly stressed while raising awareness of the complications of GS. After all, the incidence of serious neurological impairments is quite low and it can be avoided through early intervention and reasonable treatment.

## Conclusion

Despite its rarity, GS could result from mumps. Given the later recovery and the possibility of life-threatening neurological impairments owing to GS, clinicians should raise awareness of this rare complication in patients with mumps.

## Data availability statement

The original contributions presented in the study are included in the article/Supplementary material, further inquiries can be directed to the corresponding authors.

## Ethics statement

Ethical review and approval was not required for the study on human participants in accordance with the local legislation and institutional requirements. Written informed consent to participate in this study was provided by the participants' legal guardian/next of kin. Written informed consent was obtained from the individual(s) for the publication of any potentially identifiable images or data included in this article.

## Author contributions

XD, XL, and CW revised the manuscript. YS, LY, and YX drafted the manuscript and provided the figures. All authors confirmed revisions and approved the final manuscript as submitted, and agreed to be accountable for all aspects of the work.

## Funding

This study was supported by the Science Technology Support Plan Projects in Sichuan province (Grant No. 2022YFS0240).

## Conflict of interest

The authors declare that the research was conducted in the absence of any commercial or financial relationships that could be construed as a potential conflict of interest.

## Publisher's note

All claims expressed in this article are solely those of the authors and do not necessarily represent those of their affiliated organizations, or those of the publisher, the editors and the reviewers. Any product that may be evaluated in this article, or claim that may be made by its manufacturer, is not guaranteed or endorsed by the publisher.

## Abbreviations

GS: Grisel's syndrome
ENT: ear, nose, and throat
ADI: atlanto-dens interval
CRP: C-reactive protein
CT: computed tomography.
