# Supplementary material for: Grisel's syndrome associated with mumps: A case report
Source: Front Pediatr. 2022 Sep 29;10:916538. doi: 10.3389/fped.2022.916538 (PMC9557164; doi:10.3389/fped.2022.916538)
Supplement: Supplementary file 1 [file Data_Sheet_1.PDF]

CARE
case report guidelines

CARE Checklist of information to include when writing a case report

(cc) BY-NC-ND

Topic	Item	Checklist item description	Reported on Line
Title	1	The diagnosis or intervention of primary focus followed by the words "case report"	Page 1, Line 1
Key Words	2	2 to 5 key words that identify diagnoses or interventions in this case report, including "case report"	Page 2, Line 17
Abstract (no references)	3a	Introduction: What is unique about this case and what does it add to the scientific literature?	Page 3; Line 2-4
	3b	Main symptoms and/or important clinical findings	Page 3; Line 6-10
	3c	The main diagnoses, therapeutic interventions, and outcomes	Page 3; Line 11-12
	3d	Conclusion—What is the main "take-away" lesson(s) from this case?	Page 3; Line 12-14
Introduction	4	One or two paragraphs summarizing why this case is unique (**may include references**)	Page 4
Patient Information	5a	De-identified patient specific information.	Page 5; Line 2-5
	5b	Primary concerns and symptoms of the patient.	Page 5; Line 3-14
	5c	Medical, family, and psycho-social history including relevant genetic information	Page 5; Line 3-4
	5d	Relevant past interventions with outcomes	Page 5; Line 6-8
Clinical Findings	6	Describe significant physical examination (PE) and important clinical findings.	Page 5; Line 9-18
Timeline	7	Historical and current information from this episode of care organized as a timeline	Page 5; Line 3-8
Diagnostic Assessment	8a	Diagnostic testing (such as PE, laboratory testing, imaging, surveys).	Page 5; Line 14-18
	8b	Diagnostic challenges (such as access to testing, financial, or cultural)	N/A
	8c	Diagnosis (including other diagnoses considered)	Page 5; Line 19-20
	8d	Prognosis (such as staging in oncology) where applicable	Page 5; Line 20
Therapeutic Intervention	9a	Types of therapeutic intervention (such as pharmacologic, surgical, preventive, self-care)	Page 5; Line 20-21
	9b	Administration of therapeutic intervention (such as dosage, strength, duration)	Page 5; Line 20
	9c	Changes in therapeutic intervention (with rationale)	N/A
Follow-up and Outcomes	10a	Clinician and patient-assessed outcomes (if available)	Page 5; Line 21
	10b	Important follow-up diagnostic and other test results	Page 5; Line 23-24
	10c	Intervention adherence and tolerability (How was this assessed?)	N/A
	10d	Adverse and unanticipated events	N/A
Discussion	11a	A scientific discussion of the strengths AND limitations associated with this case report	Page 6; Line 17-23
	11b	Discussion of the relevant medical literature **with references**.	Page 6; Line 23-29
	11c	The scientific rationale for any conclusions (including assessment of possible causes)	Page 8; Line 7-9
	11d	The primary "take-away" lessons of this case report (without references) in a one paragraph conclusion	Page 8; Line 7-9
Patient Perspective	12	The patient should share their perspective in one to two paragraphs on the treatment(s) they received	N/A
Informed Consent	13	Did the patient give informed consent? Please provide if requested	**Yes** ☑ **No** ☐
